# Work Characteristics and Personal Social Support as Determinants of Subjective Well-Being

**DOI:** 10.1371/journal.pone.0081115

**Published:** 2013-11-19

**Authors:** Stephen A. Stansfeld, Martin J. Shipley, Jenny Head, Rebecca Fuhrer, Mika Kivimaki

**Affiliations:** 1 Centre for Psychiatry, Wolfson Institute of Preventive Medicine, Barts and the London School of Medicine and Dentistry, Queen Mary University of London, London, United Kingdom; 2 Department of Epidemiology and Public Health, University College London, London, United Kingdom; 3 Department of Epidemiology, Biostatistics and Occupational Health, McGill University, Montreal, Canada; Hunter College, City University of New York (CUNY), CUNY School of Public Health, United States of America

## Abstract

**Background:**

Well-being is an important health outcome and a potential national indicator of policy success. There is a need for longitudinal epidemiological surveys to understand determinants of well-being. This study examines the role of personal social support and psychosocial work environment as predictors of well-being in an occupational cohort study.

**Methods:**

Social support and work characteristics were measured by questionnaire in 5182 United Kingdom civil servants from phase 1 of the Whitehall II study and were used to predict subjective well-being assessed using the Affect Balance Scale (range -15 to 15, SD = 4.2) at phase 2. External assessments of job control and demands were provided by personnel managers.

**Results:**

Higher levels of well-being were predicted by high levels of confiding/emotional support (difference in mean from the reference group with low levels of confiding/emotional support  =  0.63, 95%CI 0.38–0.89, p_trend_<0.001), high control at work (0.57, 95%CI 0.31–0.83, p_trend_<0.001; reference low control) and low levels of job strain (0.60, 95%CI 0.31–0.88; reference high job strain), after adjusting for a range of confounding factors and affect balance score at baseline. Higher externally assessed work pace was also associated with greater well-being.

**Conclusions:**

Our results suggest that the psychosocial work environment and personal relationships have independent effects on subjective well-being. Policies designed to increase national well-being should take account of the quality of working conditions and factors that facilitate positive personal relationships. Policies designed to improve workplaces should focus not only on minimising negative aspects of work but also on increasing the positive aspects of work.

## Introduction

There has been a search for universal measures of health outcome that can be used to measure the impact of political policy on health. Subjective well-being has been a candidate for this [1]. There are also plans for its adoption as a potential national outcome measure to supplant Gross Domestic Product [1], [2]. Furthermore, well-being might also serve as an outcome for health services. The recent emphasis on well-being rather than sickness denotes a move away from public health outcomes associated with pathology, towards a focus on wellness and its predictors, in keeping with the WHO-inspired salutogenic approach to public health and prevention [3], [4]. Well-being has also been shown to predict good physical health and longevity [5]. The hedonic definition of subjective well-being includes subjective perceptions of moods such as happiness and cognitive judgements of life satisfaction coupled with an absence of negative feelings [6], [7].

If well-being is adopted as an outcome measure, it is important to understand the predictors of well-being. There is evidence that it is influenced by intrinsic factors such as personality, coping styles, and genetic predisposition [8], [9]. Well-being also shows complex associations with current and past health experience, personal relationships, work, leisure, housing, and the experience of education [8], [10], [11], [12], [13]. Aspects of the wider social and physical environment may also be significantly linked to well-being at the individual or collective level [14].

Classic theories, such as those expounded by Freud [15] and developed further by Erikson [16], contend that the experience of work and personal relationships are central to most people’s daily life and may have powerful influences on well-being. Both poor work environments and lack of personal social support have been shown to predict psychological distress and common mental disorder, but they may also influence well-being [8], [17]. Employment is an important contributor to well-being in terms of the resources and structure it provides and in facilitating access to social networks [18]. However, well-being *within* jobs may also be contingent on the psychosocial characteristics of the work [19]. Excessive demands, lack of control over work, poor support from managers and colleagues are all related to psychological distress [20]. The associations between work characteristics and mental ill-health are well established, but there has been less analysis of work and non-work psychosocial characteristics and well-being. There is evidence that the quality of social contacts predicts well-being in the elderly [21] and that family embeddedness and provided support predicts positive affect [22] and that social participation is related to increased well-being [8]. A better understanding of work and personal relationships as determinants of well-being requires a longitudinal perspective with adjustment for potential confounding factors such as socioeconomic status, education, health behaviours, satisfaction with housing and satisfaction with leisure time that may explain these associations [13].

We examine these associations using data from the Whitehall II Study, hypothesising that a good psychosocial work environment and high levels of personal social support will be associated with higher levels of well-being, even after taking into account other sources of life satisfaction and concurrent psychological distress. Additionally, we study associations with change in well-being by including a further adjustment for baseline well-being in order to take account of unobserved individual characteristics, such as personality traits, that may influence both exposures and well-being. We also examine the effects of work and support on well-being independent of the effects on psychological distress.

## Materials and Methods

### Ethics Statement

Ethical approval for the Whitehall II study was obtained from the University College London Medical School committee on the ethics of human research. Written informed consent was obtained from all participants.

### Participants

The Whitehall II study was established between 1985 and 1988 with a target population of all male and female civil servants, aged between 35 and 55 years, in twenty London based civil service departments [23]. 10,308 civil servants were examined in phase 1 of the study– 6,895 men and 3,413 women with a response rate of 73%, the true response rate was higher because around 4% of the invited employees had moved before the study and were not eligible for inclusion [23]. We analyzed data from phase 1 (1985-88, self-report questionnaire and screening), and phase 2 (1989, postal questionnaire, response rate 79%). The mean interval between phases 1 and 2 was 2.6 years. Our analyses are based on participants for whom complete data on covariates were available. Although most study respondents were white-collar employees, a wide range of employment grades (and salaries) from office support staff to the most senior government servants were covered.

### Well-being

Well-being was measured at phase 1 and phase 2 by the Affect Balance Scale, a ten-item scale measuring the Affect Balance Score, comprising Negative Affect (five items) subtracted from Positive Affect (five items) [24]. The range of this scale was from –15 to 15 and the observed standard deviation was 4.2. At phase 1, the Affect Balance Scale was not included in the questionnaire administered to the first 2913 participants who received an earlier version of the questionnaire.

### Work characteristics

Subjective work characteristics (decision latitude, work demands, work social support) were measured using a self-report questionnaire at phase 1, the revised version of the Job Content Instrument [19]. Cronbach’s alpha, a measure of the internal consistency, was acceptable for all scales: decision latitude (15 items): 0.84; job demands (4 items): 0.67; and social support at work (6 items): 0.79. Job strain was calculated as the decision latitude score subtracted from the work demand score; the range for the job strain score was from –87 to 83 and then it was divided into tertiles [25]. The advantage of our method over the quadrant method is that it initially uses the full range of continuous scores rather than a binary score. Job strain was also classified into categories according Karasek’s job strain model where jobs with high decision latitude and low demands were ‘low strain jobs’, those with high decision latitude and high demands were ‘active jobs’, jobs with low decision latitude and low demands were ‘passive jobs’ and those with low decision latitude and high demands were ‘high strain’ jobs [19]. Work social support included items on support from supervisors and colleagues, and clarity and consistency of information from supervisors. Items on clarity and consistency of support measured informational support from supervisors [26] and clustered with the emotional support items in principal components analysis. Work social support was divided into tertiles because of the non-normal distribution of the scores.

### Externally assessed work characteristics

Control, work pace, conflicting demands, and importance of mistakes were assessed by 140 personnel managers for participants’ jobs in 19 of 20 civil service departments using a 4-point response category on a standard form. External assessments of 710 jobs were rated by two managers: weighted kappa estimates were moderate, ranging from 0.49–0.57 [27].

### Personal social support

Perceived confiding/emotional social support received over the past 12 months was measured from the person nominated as closest on the Close Persons Questionnaire using assessments at phase 1 [28]. Negative aspects of close relationships (Negative Support) measured ‘worries, problems and stress’ and ‘negative interactions’ from the nominated closest person [28]. A measure of social networks outside the household was devised from questions about the frequency and number of contacts with relatives, friends, and social groups [29].

### Covariates

Socio-economic position was measured by a six-level civil service employment grade on the basis of salary [23]. We used a broader categorisation of employment grade: Administrative, Professional/Executive and Clerical/Support. Marital status was classified as married/cohabiting, single, widowed, divorced/separated. Education level assessed the highest level of formal education attained (education up to 16y: which is the formal school leaving age; education to age 18y; higher education post-18y), physical activity (amount of moderate/vigorous physical activity per week (none, <2.5hr, 2.5 hr moderate or 1hr vigorous)). Prior physical and mental illness was assessed at phase 1 by the self-reported presence of longstanding illness, disability, or infirmity. Life events during the last 12 months were assessed at phase 2.

### Statistical analysis

The Affect Balance score was approximately normally distributed and was treated as a continuous variable in the analyses. The longitudinal associations between the psychosocial work characteristics and personal social support measures at phase 1 and subsequent affect balance score at phase 2 (approximately 2½ years later) were investigated using least squares linear regression. Initial age-adjusted analyses in men and women separately suggested that most of the associations with well-being were similar in men and women. This was confirmed by fitting sex interaction terms in the dataset with men and women combined. Starting with adjustment for age and sex (model 0: the reference model), we fitted a series of models, each of which additionally adjusted for other covariates. Model 1 included socioeconomic factors (employment grade, education, ethnic group, and marital status); Model 2 included further adjustment for overall health status (physical activity and self-rated health) but did not include smoking status or alcohol consumption as these were found to not be associated with well-being. Model 3 additionally controlled for life events in the past year assessed at phase 2 and measures of the degree of satisfaction with the participant’s standard of living, present accommodation, and leisure time. Model 4 also included adjustment for affect balance score at phase 1. For each of the above models we present the difference in the phase 2 affect balance score, and its 95% confidence interval, for each level of exposure compared to the reference group. In addition, for the age and sex adjusted model (Model 0), we also show the age and sex adjusted least squares means of the affect balance scores at phase 2 for each level of exposure. In addition, to account for the effect of psychological distress we adjusted for General Health Questionnaire score in models 3 and 4 and also repeated the analyses in a sample who were neither General Health Questionnaire cases at phase 1 nor phase 2.

We used multiple imputation (Proc MI in SAS) to assign values for variables with missing data. The purpose was to examine potential non-response or selection bias by comparing analyses performed with and without imputation. Data were imputed for all 10308 Whitehall II participants and the imputation models included all variables in the analysis as well as the participants’ civil service department as differences in measures have been seen across departments. Multiple imputation models not including department were also conducted and gave very similar results. The multiple imputation process creates a number of copies of the data (10 copies in this case), each of which has values imputed for the missing data with an appropriate level of randomness. The average of the estimates from these ten analyses is calculated and the standard error obtained which comprises sampling variability as well as variability across the imputed samples. We used the SAS 9.2. (Cary, North Carolina, USA) statistical software to analyse the data.

## Results

The characteristics of participants at phase 1 are described in Table 1. Two thirds of the sample were men, 76% were married, and 93% were of Caucasian ethnic origin. The overall mean affect balance scale score was 3.45 (SE 0.06) at phase one and 3.33 (SE 0.06) at phase 2. The mean affect balance scale score in women was 2.95 (SE 0.12) at phase 1 and 2.88 (SE 0.12) at phase 2. In men the mean affect balance scale score at phase 1 was 3.65 (SE 0.07) and at phase 2 was 3.51 (SE 0.07). The pattern of well-being within age groups by gender at baseline and follow up showed no consistent trends by age. The association between subjectively reported psychosocial work characteristics and externally assessed work characteristics at phase 1 and well-being at phase 2 is reported in Table 2. Low subjectively reported job strain was associated with higher well-being. Subjective reports of decision authority (control over work) and skill discretion were both related to higher well-being, whereas the association of well-being with externally assessed decision authority was no longer significant after full adjustment. Low subjectively reported conflicting demands were associated with higher well-being, although low externally assessed conflicting demands were associated with low well-being; in the imputed model this was no longer significant (Table S1). High externally assessed work pace but not subjectively assessed work pace was associated with higher mean well-being scores. High work social support was associated with high levels of well-being. Tests for interaction for the difference in effects between men and women were non-significant with only those for job strain (p = 0.08) and work social support (p = 0.03) being marginally significant. These results give some reinforcement to the previous impression that the effects of the work social support measure and job strain were greater in women compared to men.

**Table 1:** Characteristics of participants at the phase 1 baseline.

Covariates		Men (N = 3663)	Women (N = 1519)	Total (N = 5182*)
		N (%)	N (%)	N (%)
**Age group**	34–39	1005 (27.4)	358 (23.6)	1363 (26.3)
	40–44	1021 (27.9)	389 (25.6)	1410 (27.2)
	45–49	739 (20.2)	345 (22.7)	1084 (20.9)
	50–56	898 (24.5)	427 (28.1)	1325 (25.6)
**Employment grade**	High	1424 (38.9)	154 (10.1)	1578 (30.5)
	Medium	1979 (54.0)	687 (45.2)	2666 (51.5)
	Low	260 (7.1)	678 (44.6)	938 (18.1)
**Ethnic group**	White	3436 (93.8)	1373 (90.4)	4809 (92.8)
	South Asian	161 (4.4)	72 (4.7)	233 (4.5)
	Black	48 (1.3)	55 (3.6)	103 (2.0)
	Other	18 (0.5)	19 (1.3)	37 (0.7)
**Marital status**	Married/cohabiting	3018 (82.4)	941 (62.0)	3959 (76.4)
	Single	471 (12.9)	327 (21.5)	798 (15.4)
	Divorced/widowed	174 (4.8)	251 (16.5)	425 (8.2)
				
**Education level**	Up to age 16	989 (27.0)	709 (46.7)	1698 (32.8)
	17 – 18	952 (26.0)	359 (23.6)	1311 (25.3)
	Over 18	1722 (47.0)	451 (29.7)	2173 (41.9)
**Self-rated health**	Very good	1413 (38.6)	410 (27.0)	1823 (35.2)
	Good	1531 (41.8)	600 (39.5)	2131 (41.1)
	Average	590 (16.1)	399 (26.3)	989 (19.1)
	Poor	116 (3.2)	100 (6.6)	216 (4.2)
	Very poor	13 (0.4)	10 (0.7)	23 (0.4)
**Longstanding illness**	No	2516 (68.7)	1030 (67.8)	3546 (68.4)
	Yes	1147 (31.3)	489 (32.2)	1636 (31.6)
**Smoking habit**	Never smoker	1766 (48.2)	820 (54.0)	2586 (49.9)
	Ex-smoker	1370 (37.4)	372 (24.5)	1742 (33.6)
	Current smoker	527 (14.4)	327 (21.5)	854 (16.5)
**Units of alcohol per wk**	None	469 (12.8)	403 (26.5)	872 (16.8)
	1–21 (M)/1–14 (F)	2517 (68.7)	962 (63.3)	3479 (67.1)
	≥ 22 (M)/≥ 15 (F)	677 (18.5)	154 (10.1)	831 (16.0)
**Life events in past year**	None	1125 (30.7)	342 (22.5)	1467 (28.3)
	1	1187 (32.4)	452 (29.8)	1639 (31.6)
	≥ 2	1351 (36.9)	725 (47.7)	2076 (40.1)
**Satisfaction with standard of living**	Dissatisfied	816 (22.3)	272 (17.9)	1088 (21.0)
	Neutral	92 (2.5)	51 (3.4)	143 (2.5)
	Satisfied	2755 (75.2)	1196 (78.7)	3951 (76.3)
**Satisfaction with present accommodation**	Dissatisfied	643 (17.6)	258 (17.0)	901 (17.4)
	Neutral	65 (1.8)	27 (1.8)	92 (1.8)
	Satisfied	2955 (80.7)	1160 (81.2)	4115 (80.8)
**Satisfaction with leisure time**	Dissatisfied	1173 (32.0)	497 (32.7)	1670 (32.2)
	Neutral	149 (4.1)	78 (5.1)	227 (4.4)
	Satisfied	2341 (63.9)	944 (62.1)	3285 (63.4)

* Participants included are those with known affect balance score at phases 1 and 2 and having no missing values on any of the covariates.

**Table 2:** Association between psychosocial work characteristics measured at phase 1 and affect balance score measured at phase 2.

Exposure			Difference in affect balance score from reference group (95% confidence interval)				
	N	Mean# (SE)	Model 0	Model 1	Model 2	Model 3	Model 4
**Conflicting demands - subjective**							
High	1501	2.77 (0.11)	0.00	0.00	0.00	0.00	0.00
Medium	2327	3.42 (0.09)	0.66 (0.39,0.93)	0.74 (0.47,1.00)	0.58 (0.32,0.85)	0.39 (0.15,0.64)	0.28 (0.05,0.50)
Low	1351	3.78 (0.11)	1.01 (0.70,1.33)	1.22 (0.91,1.54)	1.01 (0.70,1.32)	0.60 (0.31,0.89)	0.40 (0.13,0.66)
P-value for trend			<0.001	<0.001	<0.001	<0.001	0.003
**Conflicting demands – externally assessed**							
High	1701	3.61 (0.10)	0.00	0.00	0.00	0.00	0.00
Medium	1429	3.36 (0.11)	–0.25 (–0.54,0.04)	–0.13 (–0.42,0.16)	–0.05 (–0.33,0.23)	–0.06 (–0.33,0.21)	–0.07 (–0.31,0.18)
Low	1606	2.88 (0.10)	–0.73 (–1.01, –0.45)	–0.46 (–0.75, –0.16)	–0.34 (–0.63, –0.06)	–0.37 (–0.64, –0.10)	–0.33 (–0.58, –0.09)
P-value for trend			<0.001	0.002	0.02	0.008	0.008
**Work pace - subjective**							
Low	1627	3.24 (0.10)	0.00	0.00	0.00	0.00	0.00
Medium	1853	3.34 (0.10)	0.09 (–0.18,0.37)	–0.07 (–0.35,0.20)	–0.11 (–0.38,0.16)	–0.04 (–0.30,0.21)	–0.07 (–0.30,0.16)
High	1681	3.41 (0.10)	0.16 (–0.12,0.45)	–0.16 (–0.46,0.13)	–0.10 (–0.39,0.19)	0.10 (–0.17,0.38)	–0.05 (–0.30,0.19)
P-value for trend			0.27	0.28	0.51	0.45	0.67
**Work pace - externally assessed**							
Low	1444	2.92 (0.11)	0.00	0.00	0.00	0.00	0.00
Medium	2001	3.30 (0.09)	0.38 (0.10,0.66)	0.16 (–0.13,0.44)	0.16 (–0.12,0.44)	0.16 (–0.10,0.43)	0.18 (–0.06,0.42)
High	1291	3.67 (0.12)	0.75 (0.44,1.06)	0.54 (0.22,0.85)	0.46 (0.16,0.77)	0.46 (0.17,0.75)	0.41 (0.15,0.68)
P-value for trend			<0.001	<0.001	0.003	0.002	0.002
**Decision authority – subjective**							
Low	1786	2.39 (0.10)	0.00	0.00	0.00	0.00	0.00
Medium	1704	3.48 (0.10)	1.09 (0.81,1.36)	1.01 (0.73,1.30)	0.87 (0.60,1.15)	0.80 (0.54,1.07)	0.35 (0.11,0.59)
High	1673	4.19 (0.10)	1.80 (1.52,2.09)	1.70 (1.40,2.00)	1.45 (1.15,1.74)	1.24 (0.96,1.52)	0.57 (0.31,0.83)
P-value for trend			<0.001	<0.001	<0.001	<0.001	<0.001
**Decision authority - externally assessed**							
Low	1352	2.87 (0.11)	0.00	0.00	0.00	0.00	0.00
Medium	1919	3.43 (0.09)	0.56 (0.27,0.85)	0.24 (–0.07,0.54)	0.23 (–0.07,0.53)	0.30 (0.02,0.59)	0.25 (–0.01,0.51)
High	1465	3.47 (0.11)	0.60 (0.29,0.91)	0.13 (–0.21,0.47)	0.04 (–0.29,0.37)	0.14 (–0.17,0.46)	0.07 (–0.21,0.35)
P-value for trend			<0.001	0.51	0.94	0.47	0.77
**Job strain**							
Low strain	1261	4.35 (0.12)	0.00	0.00	0.00	0.00	0.00
Passive	1249	2.98 (0.12)	–1.37 (–1.70, –1.04)	–1.15 (–1.49, –0.80)	–0.95 (–1.28, –0.61)	–0.84 (–1.16, –0.53)	–0.35 (–0.64, –0.06)
Active	1557	3.50 (0.11)	–0.85 (–1.16, –0.54)	–0.88 (–1.18, –0.57)	–0.74 (–1.03, –0.44)	–0.52 (–0.80, –0.24)	–0.33 (–0.59, –0.07)
High strain	1096	2.30 (0.12)	–2.05 (–2.38, –1.71)	–1.90 (–2.23, –1.56)	–1.60 (–1.93, –1.27)	–1.23 (–1.54, –0.91)	–0.60 (–0.88, –0.31)
**Job strain - externally assessed**							
Low strain	729	3.14 (0.15)	0.00	0.00	0.00	0.00	0.00
Passive	1595	2.96 (0.10)	–0.18 (–0.55,0.19)	0.12 (–0.26,0.50)	0.20 (–0.17,0.57)	0.23 (–0.12,0.58)	0.23 (–0.09,0.54)
Active	1478	3.68 (0.11)	0.54 (0.17,0.91)	0.45 (0.08,0.82)	0.41 (0.05,0.77)	0.50 (0.16,0.83)	0.46 (0.15,0.77)
High strain	919	3.37 (0.11)	0.23 (–0.17,0.63)	0.34 (–0.06,0.75)	0.35 (–0.05,0.74)	0.31 (–0.06,0.68)	0.40 (0.06,0.73)
**Skill discretion**							
Low	1733	2.15 (0.10)	0.00	0.00	0.00	0.00	0.00
Medium	1697	3.33 (0.10)	1.17 (0.90,1.45)	1.18 (0.89,1.47)	1.09 (0.81,1.37)	0.88 (0.61,1.15)	0.41 (0.16,0.65)
High	1746	4.50 (0.10)	2.34(2.06,2.62)	2.40(2.08,2.72)	2.19(1.88,2.50)	1.93(1.63,2.22)	0.84 (0.56,1.12)
P-value for trend			<0.001	<0.001	<0.001	<0.001	<0.001
**Work social support**							
Low	1795	2.38 (0.10)	0.00	0.00	0.00	0.00	0.00
Medium	1676	3.57 (0.10)	1.19 (0.92,1.46)	1.09 (0.82,1.36)	1.00 (0.73,1.26)	0.87 (0.62,1.12)	0.50 (0.27,0.73)
High	1704	4.09 (0.10)	1.71 (1.44,1.98)	1.65 (1.38,1.92)	1.45 (1.18,1.71)	1.20 (0.95,1.46)	0.61 (0.38,0.84)
P-value for trend			<0.001	<0.001	<0.001	<0.001	<0.001

# Means are adjusted for age and sex.

Model 0  =  Adjusted for age and sex.

Model 1  =  Adjusted for age, sex, employment grade, education, ethnic group and marital status.

Model 2  =  Adjusted as for Model 1 + overall health status (physical activity and self-rated health).

Model 3  =  Adjusted as for Model 2 + life events and satisfaction with standard of living, present accommodation and leisure time.

Model 4  =  Adjusted as for Model 3 + affect balance score at Phase 1.

The analyses in models 3 and 4 were repeated adjusting for GHQ score at phase 2 to account for the effect of psychological distress confounding the association of work characteristics and well-being s S3 & S4). Associations were maintained for externally assessed conflicting demands, externally assessed work pace, and subjective decision authority, skill discretion and work social support. In the sample with GHQ cases removed at baseline and follow up, decision authority, skill discretion and work social support were still significantly related to well-being but not the adverse work characteristics (Tables S5 & S6).

The associations between personal social support at phase 1 and Affect Balance Scale score measured at phase 2 are reported in Table 3. High levels of confiding-emotional support, practical support, and network support were all related to higher levels of well-being that were maintained after full adjustment. Low negative aspects of close relationships were also consistently related to higher levels of well-being. There were no significant interactions of personal social support by sex except for negative aspects of close relationships that approach significance (p = 0.09) with larger estimated effects in women compared to men. The analyses in models 3 and 4 were repeated adjusting for GHQ score at phase 2 to account for the effect of psychological distress confounding the association of personal social support and well-being (Tables S3 & S4). Associations were maintained for confiding/emotional support, practical support and network support but were lost for negative aspects of close relationships in model 4. In the sample with GHQ cases removed at baseline and follow up the same pattern was observed (Tables S5 & S6).

**Table 3:** Association between personal social support measured at phase 1 and affect balance score measured at phase 2.

Exposure			Difference in affect balance score from reference group (95% confidence interval)				
	N	Mean# (SE)	Model 0	Model 1	Model 2	Model 3	Model 4
**Confiding/emotional support**							
Low	1538	2.19 (0.10)	0.00	0.00	0.00	0.00	0.00
Medium	1975	3.41 (0.09)	1.23 (0.96,1.50)	1.19 (0.91,1.46)	1.15 (0.88,1.41)	0.80 (0.55,1.06)	0.51 (0.28,0.74)
High	1585	4.41 (0.10)	2.22 (1.94,2.51)	2.14 (1.85,2.43)	1.94 (1.66,2.23)	1.33 (1.06,1.61)	0.63 (0.38,0.89)
P-value for trend			<0.001	<0.001	<0.001	<0.001	<0.001
**Practical support**							
Low	1656	2.66 (0.10)	0.00	0.00	0.00	0.00	0.00
Medium	1751	3.51 (0.10)	0.85 (0.57,1.12)	0.74 (0.45,1.03)	0.74 (0.46,1.02)	0.54 (0.28,0.81)	0.37 (0.13,0.61)
High	1696	3.87 (0.10)	1.21 (0.93,1.50)	1.07 (0.77,1.38)	1.10 (0.80,1.39)	0.81 (0.53,1.09)	0.42 (0.17,0.67)
P-value for trend			<0.001	<0.001	<0.001	<0.001	0.002
**Negative support**							
High	1530	2.31 (0.10)	0.00	0.00	0.00	0.00	0.00
Medium	1808	3.32 (0.10)	1.01 (0.72,1.29)	0.92 (0.64,1.21)	0.86 (0.59,1.14)	0.42 (0.15,0.69)	0.07 (–0.18,0.31)
Low	1529	4.20 (0.09)	1.89 (1.62,2.16)	1.85 (1.58,2.12)	1.67 (1.40,1.93)	0.93 (0.66,1.19)	0.34 (0.10,0.58)
P-value for trend			<0.001	<0.001	<0.001	<0.001	0.005
**Network support**							
Low	1529	2.46 (0.10)	0.00	0.00	0.00	0.00	0.00
Medium	1808	3.28 (0.10)	0.82 (0.54,1.10)	0.76 (0.48,1.04)	0.73 (0.46,1.00)	0.52 (0.26,0.78)	0.21 (–0.03,0.45)
High	1830	4.09 (0.10)	1.63 (1.35,1.91)	1.57 (1.29,1.84)	1.39 (1.17,1.67)	0.94 (0.67,1.21)	0.45 (0.20,0.69)
P-value for trend			<0.001	<0.001	<0.001	<0.001	<0.001

# Means are adjusted for age and sex.

Model 0  =  Adjusted for age and sex.

Model 1  =  Adjusted for age, sex, employment grade, education, ethnic group and marital status.

Model 2  =  Adjusted as for Model 1 + overall health status (physical activity and self-rated health).

Model 3  =  Adjusted as for Model 2 + life events and satisfaction with standard of living, present accommodation and leisure time.

Model 4  =  Adjusted as for Model 3 + affect balance score at Phase 1.

The models for work characteristics and personal social support were repeated using multiple imputation to deal with missing data and address potential selection bias (Tables S1, S2). The results were consistent with the complete case analysis, except that externally assessed conflicting demands were no longer significantly associated with well-being in the fully adjusted model.

## Discussion

Mean well-being levels were higher in men than women at both phases and declined during middle age as has been found in other studies [13]. We found that high levels of control at work, low levels of job strain, and high levels of personal social support were associated with higher levels of well-being. These associations were maintained after adjustment for affect balance score at baseline and satisfaction with standard of living, accommodation, and leisure time [30] suggesting that the psychosocial work environment and personal relationships have independent effects on subjective well-being. Externally assessed work pace was also associated with higher well-being. In this white-collar cohort, jobs with high demands also tend to be jobs with high availability of resources [20]- this combination seems to lead to increased well-being [31]. Whereas both externally and subjectively assessed decision authority were associated with greater well-being, this was not the case for conflicting demands where high subjectively assessed demands were associated with lower well-being. This result is in contrast with externally assessed demands, although the effect of externally assessed demands was no longer significant in imputed models. The results for subjectively assessed job strain were as expected: that we found the highest levels of well-being in those with ‘low job strain’- the most beneficial combination and ‘active jobs’ where there are plenty of resources to deal with job demands. In contrast, jobs which are ‘passive’ and ‘high strain’- the most adverse combination have lower well-being. What is unexpected is that the results for externally assessed job strain do not match this. Externally assessed ‘active jobs’ have the highest well-being while ‘low strain’ and ‘passive jobs’ have the lowest well-being. This ranking seems to fit more closely with the social status of jobs. Posts in the higher employment grades tend to have both higher decision latitude and high demands while ‘passive jobs’ with low demands and low decision latitude are in keeping with posts in the clerical and support grades. These results may mean that although the subjective assessments focus more on people’s own perceptions of jobs regardless of status, the externally assessed posts include aspects of the position in the organisation as well as the local working conditions.

As expected, the effects of subjectively reported work characteristics are stronger than the externally assessed work characteristics. Externally assessed work characteristics were assessed by personnel managers. The advantage of these assessments is that they could be considered ‘objective;’ they avoid the subjective response bias associated with individual’s judgement of their own jobs. The disadvantage may be that personnel managers may not be fully aware of the nature of the posts they are assessing which may weaken the associations between work characteristics and well-being. The stronger association with subjective work characteristics may reflect that the peoples’ perception of their work, rather than the objectively measured aspects of work, have stronger effects on well-being. Such perceptions are likely to partly reflect objective working conditions, but filtered through their own views of their work and relationships with fellow employees, line managers, and attitudes to their employer.

There is potential confounding by psychological distress in the association of work and personal social support with well-being. We adjusted for this in two ways by adjusting models 3 and 4 for GHQ score at phase 2 and by examining our earlier models in a sample from which GHQ cases at either phase 1 or phase 2 were removed. The first of these techniques tended to weaken the effects of adverse work characteristics but externally assessed pace and conflicting demands still showed significant effects. Using the second, perhaps more rigorous adjustment, the effects of these adverse work characteristics were no longer seen but the effects of subjective positive work characteristics: decision authority, skill discretion and work social support retained significance. An association between higher levels of control at work and well-being has also been shown in a recent cross-sectional study of a national adult population sample [32].

A similar pattern was shown for personal social support using both techniques of adjustment for psychological distress where all the positive aspects of social support still significantly predicted well-being while negative aspects of close relationships became non-significant. This could be interpreted as a form of longitudinal optimism bias where positive traits predict positive traits but it might alternatively be the case that positive aspects of work and personal relationships make people feel better when the effects of concurrent psychological distress have been excluded.

As well as hedonic definitions of subjective well-being there are also eudaimonic theories that view well-being as the realisation of human potential involving concepts such as autonomy, growth, and mastery, including aspects of successful functioning as well as subjective feelings and satisfaction. In this paper we have restricted ourselves to a narrower but more specific definition of subjective well-being [33].

A limitation of these analyses is that the data are not current, having been collected at the first and second phase of the Whitehall II Study in the latter half of the 1980’s; although the basic associations are unlikely to have changed much with time. Nevertheless, there have been large changes in the working environment in the last thirty years that could have influenced the association of work characteristics and well-being. There were gender differences in employment grade with men more likely to be in high employment grades and women more likely to be in low employment grades. However, we did see the same pattern of results when we examined men and women separately. There are some drawbacks to the measurement of work characteristics, which, although reliable, may not fully capture the complex nature of individual jobs. There are also potential limitations to generalisability from the Whitehall II study as the workforce are mainly London-based, male and middle aged. In this study jobs may not always be typical of the wider workforce- jobs with high levels of demands also tend to be high status jobs that have high levels of resources and control, Karasek’s so-called ‘active’ jobs are likely to stimulate rather inhibit well-being [19], [31]. Thus work pace and conflicting demands that are stressful and related to psychological distress in blue collar contexts may be less likely to be associated with negative consequences in this cohort. It may also be that these job demands are associated with psychological distress but not necessarily inversely associated with well-being. Thus, job strain may more accurately capture the effect of stressful jobs where the combination of low control and high demands may be associated with lower well-being. A further limitation is that well-being was measured by the Affect Balance Scale which, although a reliable scale, is no longer ‘state of the art’ for well-being measures.

Despite limitations, our findings have important implications. They suggest that policies that increase employees’ sense of control and support in the workplace are likely to lead to greater well-being [17]. In much of the debate on work-related stress there has been a focus on the negative consequences of work. This research reverses this perspective and suggests examining factors that improve the work environment and increase well-being and morale at work [34], [35]. Greater well-being may also be related to greater productivity and performance at work, increased commitment and staff retention as well as effects on health and longevity [17], [36]. Personal social support is less susceptible to the influence of social policy by its very nature. Nevertheless, it appears to be an important predictor of well-being [13]. Indirectly, government policies may influence the capacity to maintain personal relationships, through reduction of social inequalities, through housing design that promotes rather than inhibits social contacts with neighbours [37], through provision of local jobs, maternity and paternity leave, ability to have flexible working hours [38], consideration of work-life balance, and prohibitions on long working hours.

## Supporting Information

Table S1 **Association between psychosocial work characteristics measured at phase 1 and affect balance score measured at phase 2 using multiple imputation (N = 10308).**
(DOCX)

Table S2 **Association between personal social support measured at phase 1 and affect balance score measured at phase 2 using multiple imputation (N = 10308).**
(DOCX)

Table S3 **Association between psychosocial work characteristics measured at phase 1 and affect balance score measured at phase 2 including adjustment for GHQ caseness at phase 2.**
(DOCX)

Table S4 **Association between personal social support measured at phase 1 and affect balance score measured at phase 2 including adjustment for GHQ caseness at phase 2.**
(DOCX)

Table S5 **Association between psychosocial work characteristics measured at phase 1 and affect balance score measured at Phase 2 among those without GHQ caseness at Phases 1 & 2.**
(DOC)

Table S6 **Association between personal social support measured at phase 1 and affect balance score measured at phase 2 among those without GHQ caseness at phases 1 & 2.**
(DOC)
