# Supplementary material for: Work Characteristics and Personal Social Support as Determinants of Subjective Well-Being
Source: PLoS One. 2013 Nov 19;8(11):e81115. doi: 10.1371/journal.pone.0081115 (PMC3834222; doi:10.1371/journal.pone.0081115)
Supplement: Table S1 — Association between psychosocial work characteristics measured at phase 1 and affect balance score measured at phase 2 using multiple imputation (N = 10308). (DOCX) [file pone.0081115.s001.docx]

Table S1: Association between psychosocial work characteristics measured at phase 1 and affect balance score measured at phase 2 using multiple imputation (N=10308)

| **Exposure** |  | **Difference in affect balance score from reference group (95% confidence interval)** | |
| --- | --- | --- | --- |
|  |  | Adjusted for demographic factors^$^ | Fully adjusted^#^ |
|  |  |  |  |
| **Conflicting demands – subjective** | |  |  |
| High |  | 0.00 | 0.00 |
| Medium |  | 0.76 (0.55,0.98) | 0.24 (0.06,0.42) |
| Low |  | 1.17 (0.94,1.41) | 0.31 (0.06,0.57) |
| P-value for trend |  | <0.001 | 0.009 |
|  |  |  |  |
| **Conflicting demands – externally assessed** | | |  |
| High |  | 0.00 | 0.00 |
| Medium |  | -0.15 (-0.40,0.09) | -0.06 (-0.28,0.16) |
| Low |  | -0.34 (-0.63,-0.05) | -0.13 (-0.37,0.12) |
| P-value for trend |  | 0.02 | 0.41 |
|  |  |  |  |
| **Work pace - subjective** |  |  |  |
| Low |  | 0.00 | 0.00 |
| Medium |  | -0.10 (-0.33,0.14) | -0.06 (-0.28,0.16) |
| High |  | -0.08 (-0.32,0.16) | 0.04 (-0.18,0.25) |
| P-value for trend |  | 0.93 | 0.36 |
|  |  |  |  |
| **Work pace – externally assessed** | |  |  |
| Low |  | 0.00 | 0.00 |
| Medium |  | 0.24 (0.00,0.48) | 0.19 (-0.01,0.39) |
| High |  | 0.55 (0.26,0.83) | 0.38 (0.14,0.63) |
| P-value for trend |  | <0.001 | 0.002 |
|  |  |  |  |
| **Decision authority** |  |  |  |
| Low |  | 0.00 | 0.00 |
| Medium |  | 0.97 (0.77,1.18) | 0.37 (0.19,0.55) |
| High |  | 1.62 (1.39,1.85) | 0.59 (0.35,0.82) |
| P-value for trend |  | <0.001 | <0.001 |
|  |  |  |  |
| **Decision authority – externally assessed** | | |  |
| Low |  | 0.00 | 0.00 |
| Medium |  | 0.31 (0.06,0.56) | 0.20 (-0.01,0.42) |
| High |  | 0.24 (-0.07,0.56) | 0.04 (-0.25,0.34) |
| P-value for trend |  | 0.13 | 0.87 |
|  |  |  |  |
| **Job strain** |  |  |  |
| Low strain |  | 0.00 | 0.00 |
| Passive |  | -1.19 (-1.44,-0.94) | -0.41 (-0.64,-0.18) |
| Active |  | -0.94 (-1.19,-0.70) | -0.31 (-0.53,-0.09) |
| High strain |  | -1.94 (-2.21,-1.66) | -0.65 (-0.91,-0.40) |
|  |  |  |  |
| **Job strain – externally assessed** | |  |  |
| Low strain |  | 0.00 | 0.00 |
| Passive |  | -0.06 (-0.38,0.27) | 0.18 (-0.15,0.50) |
| Active |  | 0.25 (-0.07,0.56) | 0.22 (-0.09,0.54) |
| High strain |  | 0.22 (-0.18,0.62) | 0.25 (-0.14,0.65) |
|  |  |  |  |
| **Skill discretion** |  |  |  |
| Low |  | 0.00 | 0.00 |
| Medium |  | 1.32 (1.08,1.57) | 0.55 (0.30,0.80) |
| High |  | 2.39(2.11,2.67) | 0.92 (0.61,1.24) |
| P-value for trend |  | <0.001 | <0.001 |
|  |  |  |  |
| **Work social support** |  |  |  |
| Low |  | 0.00 | 0.00 |
| Medium |  | 1.11 (0.91,1.32) | 0.48 (0.29,0.66) |
| High |  | 1.76 (1.52,2.00) | 0.65 (0.43,0.86) |
| P-value for trend |  | <0.001 | <0.001 |

^$^ Adjustment as in Model 1 in tables 2 & 3 - adjusted for age, sex, employment grade, education, ethnic group and marital status

^#^ Adjustment as in Model 4 in tables 2 & 3 - adjusted for age, sex, employment grade, education, ethnic group, marital status, overall health status (physical activity and self-rated health), life events, satisfaction with: standard of living, present accommodation and leisure time, affect balance score at Phase 1
