# Supplementary material for: Work Characteristics and Personal Social Support as Determinants of Subjective Well-Being
Source: PLoS One. 2013 Nov 19;8(11):e81115. doi: 10.1371/journal.pone.0081115 (PMC3834222; doi:10.1371/journal.pone.0081115)
Supplement: Table S2 — Association between personal social support measured at phase 1 and affect balance score measured at phase 2 using multiple imputation (N = 10308). (DOCX) [file pone.0081115.s002.docx]

Table S2: Association between personal social support measured at phase 1 and affect balance score measured at phase 2 using multiple imputation (N=10308)

| **Exposure** |  | **Difference in affect balance score from reference group (95% confidence interval)** | |
| --- | --- | --- | --- |
|  |  | Adjusted for demographic factors^$^ | Fully adjusted^#^ |
|  |  |  |  |
| **Confiding/emotional support** | |  |  |
| Low |  | 0.00 | 0.00 |
| Medium |  | 1.10 (0.83,1.37) | 0.42 (0.17,0.68) |
| High |  | 2.08 (1.77,2.39) | 0.58 (0.25,0.91) |
| P-value for trend |  | <0.001 | <0.001 |
|  |  |  |  |
| **Practical support** | |  |  |
| Low |  | 0.00 | 0.00 |
| Medium |  | 0.68 (0.34,1.02) | 0.30 (-0.03,0.63) |
| High |  | 1.08 (0.76,1.41) | 0.44 (0.08,0.80) |
| P-value for trend |  | <0.001 | 0.07 |
|  |  |  |  |
| **Negative support** |  |  |  |
| High |  | 0.00 | 0.00 |
| Medium |  | 0.88 (0.58,1.19) | 0.08 (-0.21,0.36) |
| Low |  | 1.86 (1.59,2.13) | 0.42 (0.20,0.65) |
| P-value for trend |  | <0.001 | 0.001 |
|  |  |  |  |
| **Network support** |  |  |  |
| Low |  | 0.00 | 0.00 |
| Medium |  | 0.78 (0.54,1.02) | 0.22 (0.02,0.41) |
| High |  | 1.66 (1.40,1.92) | 0.52 (0.27,0.77) |
| P-value for trend |  | <0.001 | <0.001 |

^$^ Adjustment as in Model 1 in tables 2 & 3 - adjusted for age, sex, employment grade, education, ethnic group and marital status

^#^ Adjustment as in Model 4 in tables 2 & 3 - adjusted for age, sex, employment grade, education, ethnic group, marital status, overall health status (physical activity and self-rated health), life events, satisfaction with: standard of living, present accommodation and leisure time, affect balance score at Phase 1
