# Supplementary material for: Work Characteristics and Personal Social Support as Determinants of Subjective Well-Being
Source: PLoS One. 2013 Nov 19;8(11):e81115. doi: 10.1371/journal.pone.0081115 (PMC3834222; doi:10.1371/journal.pone.0081115)
Supplement: Table S3 — Association between psychosocial work characteristics measured at phase 1 and affect balance score measured at phase 2 including adjustment for GHQ caseness at phase 2. (DOCX) [file pone.0081115.s003.docx]

Table S3: Association between psychosocial work characteristics measured at phase 1 and affect balance score measured at phase 2 including adjustment for GHQ caseness at phase 2

| **Exposure** |  | **Difference in affect balance score from reference group (95% confidence interval)** | |
| --- | --- | --- | --- |
|  |  | Adjusted as for Model 3^a^ + GHQ caseness | Adjusted as for Model 4 +  GHQ caseness |
|  |  |  |  |
| **Conflicting demands - subjective** | |  |  |
| High |  | 0.00 | 0.00 |
| Medium |  | 0.17 (-0.06,0.39) | 0.10 (-0.11,0.30) |
| Low |  | 0.24 (-0.03,0.51) | 0.11 (-0.14,0.35) |
| P-value for trend |  | 0.07 | 0.38 |
|  |  |  |  |
| **Conflicting demands – externally assessed** | | |  |
| High |  | 0.00 | 0.00 |
| Medium |  | -0.01 (-0.25,0.23) | -0.02 (-0.24,0.20) |
| Low |  | -0.35 (-0.59,-0.10) | -0.31 (-0.53,-0.09) |
| P-value for trend |  | 0.006 | 0.008 |
|  |  |  |  |
| **Work pace - subjective** |  |  |  |
| Low |  | 0.00 | 0.00 |
| Medium |  | 0.17 (-0.07,0.40) | 0.11 (-0.10,0.33) |
| High |  | 0.40 (0.15,0.64) | 0.22 (0.00,0.45) |
| P-value for trend |  | 0.002 | 0.05 |
|  |  |  |  |
| **Work pace - externally assessed** | |  |  |
| Low |  | 0.00 | 0.00 |
| Medium |  | 0.00 (-0.24,0.24) | 0.03 (-0.19,0.25) |
| High |  | 0.30 (0.04,0.56) | 0.28 (0.04,0.52) |
| P-value for trend |  | 0.03 | 0.02 |
|  |  |  |  |
| **Decision authority – subjective** |  |  |  |
| Low |  | 0.00 | 0.00 |
| Medium |  | 0.56 (0.32,0.80) | 0.21(-0.02,0.43) |
| High |  | 0.93 (0.67,1.18) | 0.39 (0.16,0.63) |
| P-value for trend |  | <0.001 | <0.001 |
|  |  |  |  |
| **Decision authority - externally assessed** | | |  |
| Low |  | 0.00 | 0.00 |
| Medium |  | 0.35 (0.09,0.61) | 0.29 (0.06,0.53) |
| High |  | 0.14 (-0.14,0.43) | 0.08 (-0.18,0.34) |
| P-value for trend |  | 0.46 | 0.73 |
|  |  |  |  |
| **Job strain** |  |  |  |
| Low strain |  | 0.00 | 0.00 |
| Passive |  | -0.71 (-1.00,-0.42) | -0.31 (-0.57,-0.04) |
| Active |  | -0.32 (-0.57,-0.06) | -0.18 (-0.41,-0.06) |
| High strain |  | -0.82 (-1.10,-0.53) | -0.33 (-0.59,-0.06) |
|  |  |  |  |
| **Job strain - externally assessed** | |  |  |
| Low strain |  | 0.00 | 0.00 |
| Passive |  | 0.26 (-0.06,0.57) | 0.25 (-0.04,0.54) |
| Active |  | 0.54 (0.23,0.84) | 0.50 (0.22,0.78) |
| High strain |  | 0.29 (-0.04,0.63) | 0.36 (0.05,0.67) |
|  |  |  |  |
| **Skill discretion** |  |  |  |
| Low |  | 0.00 | 0.00 |
| Medium |  | 0.87 (0.63,1.11) | 0.46 (0.24,0.69) |
| High |  | 1.82(1.56,2.09) | 0.92 (0.66,1.17) |
| P-value for trend |  | <0.001 | <0.001 |
|  |  |  |  |
| **Work social support** |  |  |  |
| Low |  | 0.00 | 0.00 |
| Medium |  | 0.61 (0.38,0.84) | 0.33 (0.12,0.54) |
| High |  | 0.92 (0.69,1.15) | 0.45 (0.24,0.66) |
| P-value for trend |  | <0.001 | <0.001 |

^a^ Model 3 in Tables 2 & 3 = Adjusted for age, sex, employment grade, education, ethnic group and marital status, overall health status (physical activity and self-rated health), life events and satisfaction with standard of living, present accommodation and leisure time

Model 4 in Tables 2 & 3 = Adjusted as for Model 3 + affect balance score at Phase 1
