# Supplementary material for: Work Characteristics and Personal Social Support as Determinants of Subjective Well-Being
Source: PLoS One. 2013 Nov 19;8(11):e81115. doi: 10.1371/journal.pone.0081115 (PMC3834222; doi:10.1371/journal.pone.0081115)
Supplement: Table S4 — Association between personal social support measured at phase 1 and affect balance score measured at phase 2 including adjustment for GHQ caseness at phase 2. (DOCX) [file pone.0081115.s004.docx]

Table S4: Association between personal social support measured at phase 1 and affect balance score measured at phase 2 including adjustment for GHQ caseness at phase 2

| **Exposure** |  | **Difference in affect balance score from reference group (95% confidence interval)** | |
| --- | --- | --- | --- |
|  |  | Adjusted as for Model 3^a^ + GHQ caseness | Adjusted as for Model 4 +  GHQ caseness |
|  |  |  |  |
| **Confiding/emotional support** |  |  |  |
| Low |  | 0.00 | 0.00 |
| Medium |  | 0.71 (0.48,0.94) | 0.47 (0.26,0.68) |
| High |  | 1.22 (0.97,1.47) | 0.64 (0.40,0.87) |
| P-value for trend |  | <0.001 | <0.001 |
|  |  |  |  |
| **Practical support** |  |  |  |
| Low |  | 0.00 | 0.00 |
| Medium |  | 0.50 (0.25,0.73) | 0.36 (0.14,0.58) |
| High |  | 0.82 (0.57,1.07) | 0.48 (0.25,0.72) |
| P-value for trend |  | <0.001 | <0.001 |
|  |  |  |  |
| **Negative support** |  |  |  |
| High |  | 0.00 | 0.00 |
| Medium |  | 0.22 (-0.02,0.46) | -0.05 (-0.27,0.17) |
| Low |  | 0.59 (0.34,0.83) | 0.11 (-0.11,0.34) |
| P-value for trend |  | <0.001 | 0.27 |
|  |  |  |  |
| **Network support** |  |  |  |
| Low |  | 0.00 | 0.00 |
| Medium |  | 0.55 (0.31,0.78) | 0.28 (0.06,0.50) |
| High |  | 0.92 (0.68,1.16) | 0.50 (0.28,0.72) |
| P-value for trend |  | <0.001 | <0.001 |

^a^ Model 3 in Tables 2 & 3 = Adjusted for age, sex, employment grade, education, ethnic group and marital status, overall health status (physical activity and self-rated health), life events and satisfaction with standard of living, present accommodation and leisure time

Model 4 in Tables 2 & 3 = Adjusted as for Model 3 + affect balance score at Phase 1
