# Supplementary material for: Work Characteristics and Personal Social Support as Determinants of Subjective Well-Being
Source: PLoS One. 2013 Nov 19;8(11):e81115. doi: 10.1371/journal.pone.0081115 (PMC3834222; doi:10.1371/journal.pone.0081115)
Supplement: Table S5 — Association between psychosocial work characteristics measured at phase 1 and affect balance score measured at Phase 2 among those without GHQ caseness at Phases 1 & 2. (DOC) [file pone.0081115.s005.doc]

Table S5: Association between psychosocial work characteristics measured at phase 1 and affect balance score measured at Phase 2 among those without GHQ caseness at Phases 1 & 2

| **Exposure** |  |  | **Difference in affect balance score from reference group (95% confidence interval)** | | | | |
| --- | --- | --- | --- | --- | --- | --- | --- |
|  | **N** | Mean^#^ (SE) | Model 0 | Model 1 | Model 2 | Model 3 | Model 4 |
|  |  |  |  |  |  |  |  |
| **Conflicting demands - subjective** | |  |  |  |  |  |  |
| High | 729 | 4.37 (0.13) | 0.00 | 0.00 | 0.00 | 0.00 | 0.00 |
| Medium | 1380 | 4.87 (0.09) | 0.50 (0.19,0.80) | 0.55 (0.24,0.85) | 0.47 (0.17,0.77) | 0.36 (0.07,0.65) | 0.32 (0.06,0.58) |
| Low | 881 | 4.93 (0.12) | 0.56 (0.21,0.90) | 0.69 (0.34,1.03) | 0.59 (0.25,0.93) | 0.34 (0.00,0.67) | 0.19 (-0.11,0.48) |
| P-value for trend |  |  | 0.002 | <0.001 | <0.001 | 0.05 | 0.24 |
|  |  |  |  |  |  |  |  |
| **Conflicting demands – externally assessed** | | |  |  |  |  |  |
| High | 970 | 4.90 (0.11) | 0.00 | 0.00 | 0.00 | 0.00 | 0.00 |
| Medium | 816 | 4.92 (0.12) | 0.02 (-0.30,0.34) | 0.13 (-0.19,0.44) | 0.19 (-0.12,0.50) | 0.12 (-0.18,0.42) | 0.18 (-0.09,0.44) |
| Low | 937 | 4.32 (0.11) | -0.58 (-0.89,-0.27) | -0.34 (-0.65,-0.02) | -0.27 (-0.58,0.04) | -0.33 (-0.63,-0.03) | -0.18 (-0.45,0.09) |
| P-value for trend |  |  | <0.001 | 0.04 | 0.11 | 0.04 | 0.20 |
|  |  |  |  |  |  |  |  |
| **Work pace - subjective** |  |  |  |  |  |  |  |
| Low | 1040 | 4.47 (0.11) | 0.00 | 0.00 | 0.00 | 0.00 | 0.00 |
| Medium | 1153 | 4.85 (0.10) | 0.37 (0.09,0.67) | 0.23 (-0.07,0.52) | 0.18 (-0.11,0.47) | 0.20 (-0.08,0.48) | 0.14 (-0.10,0.39) |
| High | 854 | 5.02 (0.12) | 0.55 (0.23,0.86) | 0.25 (-0.07,0.58) | 0.25 (-0.07,0.57) | 0.32 (0.01,0.63) | 0.07 (-0.20,0.34) |
| P-value for trend |  |  | <0.001 | 0.12 | 0.12 | 0.04 | 0.59 |
|  |  |  |  |  |  |  |  |
| **Work pace - externally assessed** | |  |  |  |  |  |  |
| Low | 817 | 4.39 (0.12) | 0.00 | 0.00 | 0.00 | 0.00 | 0.00 |
| Medium | 1013 | 4.84 (0.10) | 0.45 (0.14,0.76) | 0.21 (-0.11,0.53) | 0.21 (-0.10,0.52) | 0.19 (-0.11,0.49) | 0.08 (-0.19,0.34) |
| High | 1060 | 4.85 (0.12) | 0.46 (0.12,0.80) | 0.22 (-0.12,0.56) | 0.17 (-0.17,0.51) | 0.22 (-0.11,0.55) | 0.07 (-0.22,0.36) |
| P-value for trend |  |  | 0.007 | 0.21 | 0.34 | 0.19 | 0.63 |
|  |  |  |  |  |  |  |  |
| **Decision authority – subjective** |  |  |  |  |  |  |  |
| Low | 910 | 3.91 (0.11) | 0.00 | 0.00 | 0.00 | 0.00 | 0.00 |
| Medium | 1083 | 4.87 (0.11) | 0.97 (0.66,1.28) | 0.84 (0.53,1.16) | 0.74 (0.43,1.05) | 0.65 (0.35,0.95) | 0.30 (0.03,0.57) |
| High | 865 | 5.40 (0.10) | 1.49 (1.18,1.80) | 1.34 (1.01,1.67) | 1.20 (0.87,1.53) | 1.06 (0.74,1.38) | 0.44 (0.16,0.72) |
| P-value for trend |  |  | <0.001 | <0.001 | <0.001 | <0.001 | 0.003 |
|  |  |  |  |  |  |  |  |
| **Decision authority - externally assessed** | | |  |  |  |  |  |
| Low | 775 | 4.32 (0.13) | 0.00 | 0.00 | 0.00 | 0.00 | 0.00 |
| Medium | 1083 | 4.96 (0.10) | 0.64 (0.32,0.96) | 0.30 (-0.04,0.63) | 0.28 (-0.05,0.61) | 0.26 (-0.06,0.58) | 0.26 (-0.02,0.53) |
| High | 865 | 4.73 (0.12) | 0.41 (0.07,0.75) | -0.10 (-0.46,0.27) | -0.19 (-0.55,0.17) | -0.16 (-0.51,0.19) | -0.16 (-0.46,0.15) |
| P-value for trend |  |  | 0.03 | 0.44 | 0.19 | 0.24 | 0.20 |
|  |  |  |  |  |  |  |  |
| **Job strain** |  |  |  |  |  |  |  |
| Low strain | 853 | 5.36 (0.12) | 0.00 | 0.00 | 0.00 | 0.00 | 0.00 |
| Passive | 737 | 4.36 (0.13) | -1.00 (-1.34,-0.65) | -0.76 (-1.12,-0.40) | -0.62 (-0.97,-0.26) | -0.55 (-0.89,-0.20) | -0.05 (-0.36,0.25) |
| Active | 874 | 4.98 (0.12) | -0.38 (-0.70,-0.06) | -0.37 (-0.69,-0.05) | -0.29 (-0.61,-0.03) | -0.20 (-0.50,0.11) | -0.04 (-0.31,0.23) |
| High strain | 517 | 3.99 (0.15) | -1.37 (-1.74,-1.00) | -1.17 (-1.54,-0.80) | -1.01 (-1.38,-0.64) | -0.77 (-1.13,-0.41) | -0.28 (-0.60,0.04) |
|  |  |  |  |  |  |  |  |
| **Job strain - externally assessed** | |  |  |  |  |  |  |
| Low strain | 454 | 4.41 (0.16) | 0.00 | 0.00 | 0.00 | 0.00 | 0.00 |
| Passive | 911 | 4.53 (0.11) | 0.13 (-0.26,0.52) | 0.48 (0.08,0.88) | 0.52 (0.12,0.91) | 0.56 (0.18,0.94) | 0.36 (0.03,0.70) |
| Active | 830 | 5.11 (0.12) | 0.69 (0.31,1.09) | 0.62 (0.23,1.01) | 0.58 (0.19,0.96) | 0.67 (0.30,1.04) | 0.40 (0.08,0.73) |
| High strain | 524 | 4.64 (0.15) | 0.23 (-0.21,0.66) | 0.40 (-0.03,0.83) | 0.45 (0.02,0.87) | 0.48 (0.07,0.89) | 0.34 (-0.02,0.70) |
|  |  |  |  |  |  |  |  |
| **Skill discretion** |  |  |  |  |  |  |  |
| Low | 938 | 3.46 (0.11) | 0.00 | 0.00 | 0.00 | 0.00 | 0.00 |
| Medium | 982 | 4.85 (0.11) | 1.40 (1.09,1.70) | 1.39 (1.08,1.70) | 1.34 (1.03,1.64) | 1.18 (0.88,1.48) | 0.71 (0.44,0.98) |
| High | 1068 | 5.84 (0.10) | 2.38(2.07,2.68) | 2.41(2.07,2.75) | 2.30(1.96,2.63) | 2.12 (1.79,2.45) | 0.99 (0.68,1.29) |
| P-value for trend |  |  | <0.001 | <0.001 | <0.001 | <0.001 | <0.001 |
|  |  |  |  |  |  |  |  |
| **Work social support** |  |  |  |  |  |  |  |
| Low | 891 | 4.13 (0.11) | 0.00 | 0.00 | 0.00 | 0.00 | 0.00 |
| Medium | 980 | 4.83 (0.11) | 0.70 (0.39,1.01) | 0.59 (0.29,0.90) | 0.55 (0.24,0.85) | 0.54 (0.25,0.83) | 0.26 (0.00,0.51) |
| High | 1116 | 5.21 (0.10) | 1.08 (0.78,1.38) | 1.01 (0.72,1.31) | 0.95 (0.65,1.24) | 0.85 (0.56,1.13) | 0.37 (0.12,0.63) |
| P-value for trend |  |  | <0.001 | <0.001 | <0.001 | <0.001 | <0.001 |

^#^ Means are adjusted for age and sex

Model 0 = Adjusted for age and sex

Model 1 = Adjusted for age, sex, employment grade, education, ethnic group and marital status

Model 2 = Adjusted as for Model 1 + overall health status (physical activity and self-rated health)

Model 3 = Adjusted as for Model 2 + life events and satisfaction with standard of living, present accommodation and leisure time

Model 4 = Adjusted as for Model 3 + affect balance score at Phase 1
