# Supplementary material for: Work Characteristics and Personal Social Support as Determinants of Subjective Well-Being
Source: PLoS One. 2013 Nov 19;8(11):e81115. doi: 10.1371/journal.pone.0081115 (PMC3834222; doi:10.1371/journal.pone.0081115)
Supplement: Table S6 — Association between personal social support measured at phase 1 and affect balance score measured at phase 2 among those without GHQ caseness at phases 1 & 2. (DOC) [file pone.0081115.s006.doc]

Table S6: Association between personal social support measured at phase 1 and affect balance score measured at phase 2 among those without GHQ caseness at phases 1 & 2

| **Exposure** |  |  | **Difference in affect balance score from reference group (95% confidence interval)** | | | | |
| --- | --- | --- | --- | --- | --- | --- | --- |
|  | **N** | Mean^#^ (SE) | Model 0 | Model 1 | Model 2 | Model 3 | Model 4 |
|  |  |  |  |  |  |  |  |
| **Confiding/emotional support** |  |  |  |  |  |  |  |
| Low | 825 | 3.62 (0.11) | 0.00 | 0.00 | 0.00 | 0.00 | 0.00 |
| Medium | 1126 | 4.82 (0.10) | 1.20 (0.90,1.50) | 1.16 (0.86,1.46) | 1.11 (0.81,1.41) | 0.84 (0.55,1.14) | 0.47 (0.21,0.74) |
| High | 991 | 5.73 (0.11) | 2.11 (1.81,2.42) | 2.03 (1.72,2.35) | 1.91 (1.59,2.22) | 1.53 (1.22,1.84) | 0.70 (0.41,0.98) |
| P-value for trend |  |  | <0.001 | <0.001 | <0.001 | <0.001 | <0.001 |
|  |  |  |  |  |  |  |  |
| **Practical support** |  |  |  |  |  |  |  |
| Low | 916 | 4.14 (0.11) | 0.00 | 0.00 | 0.00 | 0.00 | 0.00 |
| Medium | 1030 | 4.90 (0.11) | 0.76 (0.45,1.06) | 0.65 (0.34,0.97) | 0.63 (0.32,0.94) | 0.50 (0.39,0.55) | 0.36 (0.09,0.62) |
| High | 999 | 5.26 (0.11) | 1.11 (0.81,1.42) | 0.94 (0.61,1.27) | 0.91 (0.59,1.24) | 0.75 (0.20,0.80) | 0.35 (0.07,0.63) |
| P-value for trend |  |  | <0.001 | <0.001 | <0.001 | <0.001 | 0.02 |
|  |  |  |  |  |  |  |  |
| **Negative support** |  |  |  |  |  |  |  |
| High | 688 | 4.07 (0.13) | 0.00 | 0.00 | 0.00 | 0.00 | 0.00 |
| Medium | 923 | 4.64 (0.11) | 0.57 (0.23,0.90) | 0.40 (0.07,0.74) | 0.35 (0.02,0.68) | 0.11 (-0.22,0.43) | -0.24 (-0.53,0.04) |
| Low | 1326 | 5.27 (0.09) | 1.20 (0.88,1.51) | 1.08 (0.76,1.39) | 0.97 (0.66,1.28) | 0.56 (0.25,0.87) | 0.08 (-0.20,0.35) |
| P-value for trend |  |  | <0.001 | <0.001 | <0.001 | <0.001 | 0.29 |
|  |  |  |  |  |  |  |  |
| **Network support** |  |  |  |  |  |  |  |
| Low | 853 | 4.05 (0.12) | 0.00 | 0.00 | 0.00 | 0.00 | 0.00 |
| Medium | 1012 | 4.71 (0.11) | 0.66 (0.35,0.97) | 0.57 (0.27,0.88) | 0.56 (0.26,0.86) | 0.40 (0.10,0.69) | 0.09 (-0.17,0.35) |
| High | 1116 | 5.35 (0.10) | 1.30 (1.00,1.60) | 1.19 (0.89,1.49) | 1.10 (0.79,1.40) | 0.82 (0.52,1.11) | 0.36 (0.10,0.63) |
| P-value for trend |  |  | <0.001 | <0.001 | <0.001 | <0.001 | 0.005 |

^#^ Means are adjusted for age and sex

Model 0 = Adjusted for age and sex

Model 1 = Adjusted for age, sex, employment grade, education, ethnic group and marital status

Model 2 = Adjusted as for Model 1 + overall health status (physical activity and self-rated health)

Model 3 = Adjusted as for Model 2 + life events and satisfaction with standard of living, present accommodation and leisure time

Model 4 = Adjusted as for Model 3 + affect balance score at Phase 1
